# Does exposure type impact differentially over time on the development of mental health disturbances after a technological disaster?

**DOI:** 10.1186/s13690-015-0066-z

**Published:** 2015-04-13

**Authors:** Erik De Soir, Ann Versporten, Emmanuelle Zech, Herman Van Oyen, Jacques Mylle, Rolf Kleber, Onno van der Hart

**Affiliations:** Department of Scientific and Technological Research, Royal Higher Institute for Defense, Avenue de la Renaissance 30, B-1000 Brussels, Belgium; Vaccine & Infectious Disease Institute (VAXINFECTIO), Faculty of Medicine and Health Science, Laboratory of Medical Microbiology, University of Antwerp, Universiteitsplein 1, B-2610 Antwerp, Belgium; Faculty of psychology and educational sciences, Research Center for Health and Psychological Development, Place du Cardinal Mercier, Université catholique de Louvain, 10, BE-1348 Louvain la Neuve, Belgium; Scientific Institute of Public Health, Direction Public Health and Surveillance, J. Wytsmanstreet 14, B-1050 Brussels, Belgium; Department of Behavioral Sciences, Royal Military Academy, Avenue de la Renaissance 30, B-1000 Brussels, Belgium; Faculty of Social Sciences, Department of Clinical and Health Psychology, Utrecht University, Utrecht, The Netherlands

**Keywords:** Technological disaster, Mental health disturbances, Disaster survivors

## Abstract

**Background:**

A longitudinal study was conducted in order to assess the impact of the Ghislenghien disaster (July 30th, 2004) on physical, mental and social health in the affected population. The present study explored the risk for the development of four types of mental health disturbances (MHD) due to exposure to different aspects of this technological disaster in comparison with data obtained from previous health surveys among the population of the same province.

**Methods:**

Surveys were conducted 5 months (T1) and 14 months (T2) after the disaster. Potential adult victims (≥15 years) were included (n = 1027 and 579 at T1 and T2 respectively). The “Symptom Checklist-90-Revised” (SCL-90-R) has been used in order to compute actual prevalence rates of somatization-, depression-, anxiety- and sleeping disturbances for three defined exposure categories: direct witnesses who have seen human damage (SHD), direct witnesses who have not seen human damage (NSHD) and indirect witnesses (IW). Those prevalence rates were compared with overall rates using the inhabitants of the province of Hainaut (n = 2308) as reference population. A mental health co-morbidity index was computed. Relative risks were estimated using logistic regression models.

**Results:**

Prevalence rates of the four MHD were much higher for the SHD than for the other exposure groups, at T1 and T2. Moreover, NSHD and IW had no increased risk to develop one of the 4 types of MHD compared to the reference population. The SHD had at T1 and T2 good 5-times a higher risk for somatization, about 4-times for depression and sleeping disorders, and 5- to 6-times for anxiety disorders respectively. Further, they suffered 13 times, respectively 17 times more from all mental disorders together.

**Conclusions:**

The present study calls attention to the fact that mental health problems disturbances are significantly more prevalent and long-lasting among survivors who have directly been exposed to human damage.

## Background

People exposed to potentially traumatic events in the context of a disaster often suffer from a wide range of psychological symptoms including anxiety-, depression-, and posttraumatic stress symptoms as well as physical symptoms such as somatization [1-3]. Disaster survivors are often difficult to define and the denominator is usually unclear in the direct aftermath of disaster. For this reason, disaster research often focuses on the residents of the official disaster area [4] or survivors who had to be relocated after the disaster [5]. In other cases [6], victims are identified through the medical chain and as a function of their medical condition. It is also well known that there exists a relationship between the severity of exposure and the mental health condition, called dose–response relationship, which could serve as a basis for victims classification. Severe exposure such as threat of life, the confrontation with injury and human losses or severe initial stress reactions may be considered as event-specific risk factors [7-9].

On July 30th 2004, an accidental leakage in a high pressure gas pipe, which passed under the industrial zone of Ghislenghien (Belgium) exploded and instantly killed 24 people by the blast of the explosion. From the first fire crew, only two firefighters survived the initial blast and 132 people were injured. When the first crew of firefighters arrived on-scene, an enormous explosion took place: the heat of the fire was felt up to two kilometers from the explosion site. An impressive column of fire rose into the air and the heat was felt up to two kilometers away from the explosion site. Debris from the gas pipe and buildings was projected up to six kilometers away from the epicenter; up to 16 km from the explosion, air vibrations were registered. Hundreds of fire, rescue and police personnel rushed to the disaster area and all the hospitals in the region received numerous victims. A wide area was affected by the largest technological disaster that Belgium ever knew since the mine disaster of Bois du Casier in Marcinelles in which a fire caused the death of 136 Italian and 95 Belgian mineworkers (1956).

Predictors of the intensity of the PTSD symptoms among adult survivors of the Ghislenghien disaster have already been described in De Soir et al. [10]. The kind of exposure to the disaster, in particular, the degree to which life threat was experienced, was a predictor of the severity of PTSD symptoms. Survivors were classified in three main categories: victims who had been directly exposed to the disaster and direct witnesses (primary victims), indirect witnesses (secondary victims) i.e. who had been exposed to the disaster by the intermediate of the affective proximity to a victim, and, people who could have been exposed directly to the disaster [10].

This article aims to assess the impact of the Ghislenghien gas explosion at two different points in time on the prevalence rates of four mental health disorders (MHD), namely somatization-, depression-, anxiety- and sleeping disorders, in a population affected by the disaster depending on three types of exposure. In addition, the prevalence rates will be compared with a Belgian reference population, enabling the quantification of the risk of having a MHD that can be attributed to the disaster.

Since the current literature indicates a dose–response relationship between exposure and mental health condition a classification of exposure types has been set up. Categories are have seen human damage (SHD), not have seen human damage (NSHD) and indirect witnessing (IW)

## Methods

### Procedures

#### Study population

The target group was composed of all potential victims of the Ghislenghien gas explosion. They included 1) residents of surrounding villages living up to maximum 5 km from the explosion epicenter near the industrial site and 2) employees, whether present or not, of 4 companies located on the industrial ground of Ghislenghien, as well as 3) their family members, including children from 8 to 14 years old. All the above subjects were connected with the disaster through a geographical or professional proximity as well as connections through relatives. Participation to the study was voluntary. To compare with the reference population (see below), only adult persons – i.e. aged 15 years and older - have been selected for this paper.

#### Study design

Aside of the classic socio-demographic data, five mental health indicators were measured at two time points. The first was in December 2004, 5 months after the disaster (T1). A follow-up assessment was carried out in September 2005, 14 months after the disaster (T2), enabling the evaluation of changes in health approximately one year after the disaster. As such, 1027 people (49.8% men, 50.2% women) agreed to participate in the study at T1 and 579 people (48.9% men, 51.1% women) at T2. The response rate at household level at T1 and T2 was 18% (n = 607 families) and 56% (n = 338 families). Ages ranged from 15 to 92 years with a mean age of 44.98 years (Se = 0.52). More details on the study design can be found in Versporten et al. [1].

#### Reference population

The current study used the same mental health instruments as the 2001 Belgian National Health Interview Survey, enabling us to use those data as a reference [11]. Ghislenghien is located in the province of Hainaut in Belgium. For reasons of closest comparability it was decided to include data of persons aged 15 years and older from the province of Hainaut (n = 2308), rather than those of the general Belgian population.

#### Exposure classification of victims

People described their degree of exposure to the disaster in the questionnaire by selecting among three exposure categories according to the individual’s proximity to the disaster.

1. The first category encompasses the direct witnesses who have seen human damage (SHD) (n = 84). Those persons were ‘active witnesses’ of the disaster i.e. they witnessed the deceased or severly burnt victims, tried to offer their help and were directly exposed to effects of the explosion as they were present on site at the moment of the disaster. They were expected to be more prone to adverse health consequences due to the witnessing of grotesque scenes at the site and the life treat they have experienced [12].
2. The second category are the direct witnesses who have not seen human damage (NSHD) (n = 597). They were ‘passive witnesses’ of the explosion such as local residents living in the surrounding communities up to 5 kilometers from the industrial site; they had heard, seen, smelled and experienced the disaster from a distance.
3. The third category concerns the indirect witnesses (IW) (n = 346). They have been indirectly exposed to the disaster through an affective proximity with a SHD or a NSHD. They were family members or colleagues of deceased or wounded persons. (e.g. co-workers not present on site, partner of a SHD).

A detailed overview of the defined categories, including their response rates at both time points, have been reported in Versporten et al. [1].

#### Mental health assessment

Mental health was assessed at the T1 and T2 using the depression-, anxiety-, disturbances of sleep and somatization symptoms subscales of the “Symptom Checklist-90-Revised” (SCL-90-R) to detect cases for one or more of the above and measure their intensity [10]. This self-report checklist inquires the current psychological state during the preceding week without making reference to the normal state. In this way, chronic problems are taken into account. A total score equals the sum of item scores (0 to 4), divided by the number of items in the subscale considered. Cases with more than 4 items missing on a given subscale were excluded for analysis. A threshold of 2 (SCL-score 0–1 versus 2–4) was used to assign the respondents to a group with - or a group without substantial disturbance:

- Somatization is defined by chronic complaints of widespread physical symptoms across multiple organ systems [12]. It refers to the development of physical symptoms for which no organic cause is found but which could have a psychological cause [13] (12-item scale);
- Depression is defined as the common concept of the ‘depressive syndrome’ with important characteristics as there are changes in good spirits, decline of energy, decrease of everyday activity, less capacity of oneself feeling fine, lack of interest, losing one’s concentration and feeling oneself inexplicably tired (13-item scale);
- Anxiety covers an emotional component (concern, fear, terror, etc.) as well as a physical one (tensed muscles, trembling, dry mouth, sweating, stomachache, diarrhea, etc.) (10-item scale);
- Disturbances of sleep encompassed problems to fall asleep, to wake up and the quality of sleep (3-item scale).

To compare our data with existing data on mental health disorders in the reference population, a co-morbidity scale was created : 0 up to 4 mental health disorders (MHD), 1 to 4 versus 0 MHD, and 1 to 3 or 4 versus 0 MHD respectively (see below).

### Data analysis

Prevalence rates of somatization-, depression-, anxiety- and sleeping disturbances as well as a mental health co-morbidity index were computed for the three defined exposure categories and compared with the reference population.

Multivariate logistic regression models and multinomial logistic regression models adjusting for age (categorized in 6 categories: 15–24, 25–34, 35–44, 45–54, 55–64, >65 years), sex and educational level (lower secondary or less education versus higher secondary education or university level) were used. Relative risks for mental health disorders, compared to the reference population, were computed. Finally, a proportional odds model was used. This model takes into account the order of the outcome variable, namely the number of MHD reported. The latter results are less precise but more concise than working at the symptom/cluster level. Computations were performed with SPSS 16.0 and STATA 8.

## Results

**Figure 1:**
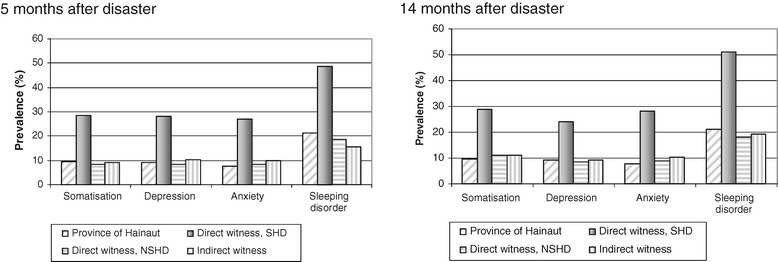
**Prevalence of mental disturbances by type of exposition at 5 and 14 months after the disaster as compared with data of the province of Hainaut (Health Interview Survey 2001).**

The prevalence rates of the four examined MHD were much higher for the SHD (28 to 48 %) than for the NSHD, the IW and the reference group (10 to 20 %) at T1. Those figures remained more or less the same at T2 except a slight reduction in prevalence for depression (see Figure 1).

Logistic regressions gave evidence of 4- to 5 times higher rates among the SHD as compared to the reference population at T1 in all four MHD. Those rates slightly increased by 0.3 to 0.7 at T2 except for sleeping disorders (decrease by 0.3). Overall, NSHD and IW did not differ from the reference group at T1 nor at T2 (see Table 1).

**Table 1:** **Odds ratios of the 4 mental health disturbances by exposure as compared to the province of Hainaut adjusted for sex, age and educational level 5 and 14 months after the disaster**

			**Depression**		**Anxiety**		**Sleeping disturbance**	
	**OR (95%CI)**		**OR (95%CI)**		**OR (95%CI)**		**OR (95%CI)**	
	**5 months**	**14 months**	**5 months**	**14 months**	**5 months**	**14 months**	**5 months**	**14 months**
Province Hainaut (Ref.)	1	1	1	1	1	1	1	1
Direct witness, SHD	5.2 (3.0-9.2)	5.6 (2.7-11.6)	4.3 (2.4-7.7)	4.6 (2.2-9.6)	5.0 (2.8-8.9)	5.9 (2.9-12.2)	4.6 (2.9-7.3)	4.2 (2.3-7.9)
Direct witness, NSHD	0.9 (0.6-1.4)	1.1 (0.7-1.7)	0.8 (0.6-1.2)	0.8 (0.5-1.3)	1.0 (0.7-1.5)	1.1 (0.7-1.7)	1.3 (0.9-1.6)	0.8 (0.5-1.1)
Indirect witness	1.3 (0.8-2.1)	1.7 (0.9-2.9)	1.3 (0.9-2.1)	1.2 (0.7-2.1)	1.2 (0.8-1.9)	1.6 (0.9-2.7)	1.5 (1.1-1.9)	0.9 (0.6-1.5)
Sex (women vs men)	1.2 (1.1-1.4)	1.3 (1.2-1.5)	1.1 (0.9-1.2)	1.1 (1.0-1.2)	0.9 (0.9-1.0)	0.9 (0.9-1.1)	1.2 (1.1-1.3)	1.2 (1.1-1.3)
Age (by 10 years)	2.0 (1.5-2.7)	2.0 (1.4-2.8)	2.1 (1.5-2.8)	2.3 (1.6-3.2)	2.2 (1.6-3.0)	2.1 (1.5-3.0)	1.2 (0.9-1.4)	1.5 (1.2-1.9)
Educational level (low vs high)	1.6 (1.1-2.1)	1.6 (1.1-2.2)	1.6 (1.2-2.2)	1.4 (1.0-2.0)	1.6 (1.1-2.2)	1.4 (0.9-2.0)	1.4 (1.1-1.7)	1.3 (1.0-1.7)

SHD = Seen human damage, NSHD = Not seen human damage.

Total N at 5 months = 2184 subjects (Ghislenghien n = 966; Hainaut n = 1218); Total N at 14 months = 1708 subjects (Ghislenghien n = 492; Hainaut n = 1216).

Multilogistic regression model.

**Figure 2:**
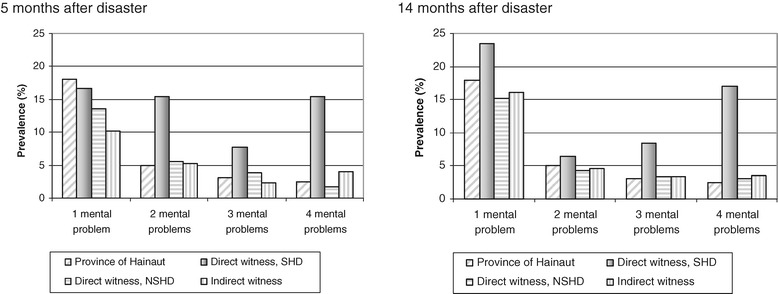
**Prevalence of the number of mental disturbances by type of exposition at 5 and 14 months after the disaster as compared with data of the province of Hainaut (HIS 2001).**

The co-morbidity prevalence was much higher among SHD compared to the other groups, at T1, the most striking difference being for 4MHD (see Figure 2): Some 15.% of the SHD versus only 1.7% of the NSHD and 4.0% of the IW at T1. At T2, the are even 17.0% of the SHD with four MHD, while the prevalence in the other categories did not change. The rates of having 2 or 3 MHD decreased at T2 as compared to T1 for the SHD but remained stable for all other groups. Moreover the prevalence of having only 1 MHD increased in all victim categories at T1 but most for the SHD.

Table 2 presents the cumulative frequency distribution of having 1 up to 4 MHD against the prevalence of persons without any MHD. At T1, 55.2% of the SHD, 24.7% of the NSHD and 21.8% of the IW, had at least one specific recent mental problem as compared to 28.4% in the reference population. At T2, this amount remained the same for the SHD, slightly increased in the NSHD and approaches the prevalence rate of the reference population in the IW.

**Table 2:** **Distribution of cumulative prevalence (%) of having 1 up to 4 mental disturbances versus persons without mental disturbance at 5 and 14 months after the disaster**

	**No mental disturbances**		**Having 1–4 mental disturbances**	
Province of Hainaut	71.6		28.4	
	5 months		14 months	
Direct witness SHD	44.8	44.7	5 months	14 months
Direct witness NSHD	75.3	74.2	55.2	55.3
Indirect witness	78.2	72.4	24.7	25.8

SHD = Seen human damage, NSHD = Not seen human damage.

Three different models were employed to test whether the people affected by the Ghislenghien disaster had a higher risk to suffer from one or more MHD as compared to the reference population. Odds ratios were computed for the 3 exposure groups. People with no mental health disorder were taken as reference group (see Table 3). First, a multivariate logistic regression model calculated stratum specific estimates of having 1 to 4 MHD, adjusted for sex, age and educational level. The results indicated that SHD had a 3.6 times higher risk of having 1 or more MHD at T1. This ratio remained the same at T2. NSHD and IW do not differ from the reference population (OR around 1). Second, a multinomial logistic regression model revealed odds ratios of having 1 to 3 MHD respectively 4 MHD by exposure type as compared to the reference population. The OR for 4 MHD in the SHD group is 12.8 at T1 and increases to 16.9 at T2. It should however be noted that large confidence intervals were found, probably due to the limited number of cases within the SHD group (n = 84 at T1 and n = 47 at T2). The risk of having all 4 MHD in the NSHD is about 1 but about 2 in the IW. Finally, a proportional odds model allowed to estimate the odds ratio of having 1 MHD more in the victims population than the reference population. SHD had a proportional odd of about 5 at T1. In other words, the chances of going, for example, from having 2 MHD to at least 3 or 4 MHD are 5 times higher in the SHD group. Similar results were found at T2 for all groups.

**Table 3:** **Odds ratios of having 1 up to 4 MHD (mental health disturbances); 1 up to 3 MHD/4 MHD; and odds ratios of being in a higher numbered category (having 1 MHD more) by exposure type as compared to the province of Hainaut adjusted for sex, age and educational level at 5 and 14 months after the disaster**

	**1- 4 MHD** ^**1**^		**1 - 3 MHD** ^**2**^		**4 MHD** ^**2**^		**Odds ratios of being in a higher numbered category** ^**3**^	
	**OR (95% CI)**		**OR (95% CI)**		**OR (95% CI)**		**OR (95% CI)**	
	**5 months**	**14 months**	**5 months**	**14 months**	**5 months**	**14 months**	**5 months**	**14 months**
Province Hainaut (Ref.)	1	1	1	1	1	1	1	1
Direct witness, SHD	3.6 (2.3-5.9)	3.6 (1.2-6.7)	2.8 (1.7-4.7)	2.5 (1.3-5.2)	12.8 (5.7-28.4)	16.9 (6.2-45.9)	4.7 (2.9-7.4)	4.9 (2.7-9.0)
Direct witness, NSHD	0.9 (0.7-1.1)	0.8 (0.6-1.1)	0.8 (0.7-1.1)	0.8 (0.6-1.1)	0.7 (0.3-1.4)	1.0 (0.4-2.5)	0.9 (0.7-1.1)	0.8 (0.6-1.1)
Indirect witness	0.9 (0.6-1.2)	1.1 (0.8-1.6)	0.7 (0.5-1.0)	1.0 (0.7-1.5)	1.8 (0.9-3.6)	1.8 (0.6-4.9)	0.9 (0.7-1.3)	1.2 (0.8-1.7)
Sex (women vs men)	1.5 (1.2-1.8)	1.7 (1.4-2.1)	1.5 (1.2-1.8)	1.6 (1.3-2.1)	2.6 (1.5-4.6)	2.6 (1.4-5.0)	1.5 (1.2-1.8)	1.6 (1.3-2.0)
Age (by 10 years)	1.2 (1.1-1.2)	1.2 (1.1-1.3)	1.2 (1.1-1.2)	1.2 (1.1-1.3)	1.1 (0.9-1.3)	1.2 (0.9-1.4)	1.2 (1.1-1.2)	1.2 (1.1-1.3)

SHD = Seen human damage, NSHD = Not seen human damage.

^1^Multivariate logistic regression model.

^2^Multinomial logistic regression model.

^3^Proportional odds model (ordinal cumulative logit model).

Use of weighted data for the reference population, the province of Hainaut, weight for the Ghislenghien population =1.

The reference category is ‘having no MHD’.

Quest.1: Ghislenghien n = 966 (direct witness SHD: n = 79, direct witness NSHD: n = 559, indirect witness: n = 328), Province of Hainaut n = 1231.

Quest.2: Ghislenghien n = 499 (direct witness SHD: n = 43, direct witness NSHD: n = 290, indirect witness: n = 166), Province of Hainaut n = 1231.

## Discussion

Our findings indicate that mental health disturbances were more prevalent in the population that was closely involved in the Ghislenghien disaster as compared to the more distant groups and to the reference population, who do not differ among each other . This is on the one hand indicative of a dose–response relationship but on the other hand of a floor effect. Moreover, the direct witnesses who had seen human damage (SHD) had significantly higher prevalence rates of recent somatization-, depression-, anxiety- and sleeping disturbances both at T1 and T2. In addition, the very high percentage of co-morbidity among the SHD is striking. Significantly more often they suffered from the four mental problems together as compared to the reference population and the other victim groups. Similar results were found at T2, suggesting that, for these victims, the impact of the disaster on mental health had not been resolved over time. This is not consistent with some studies showing a decreasing level of depression, anxiety and PTSD over time [14,15] while other studies show that certain symptoms continue to exist many years after [16].

An individual response to a disaster depends on event factors as well as individual factors. Our results confirm that physical proximity to the disaster (event factor) was a predominant criterion for the observed psychological impact [17,18] because people who have been exposed to the Ghislenghien disaster in a less drastic way (NSHD and IW) did not show the above-mentioned mental health effects. Concerning the eventual effect of individual factors, we controlled for sex, age and educational level in our analyses. Our study confirms the impact of those socio-demographic variables: women are about 1.5 times more prone to adverse health effects than men, just as younger people are more affected than elder ones (OR 2) and lower educated subjects more than higher educated OR. 1.6). Furthermore we could not take into account the history or the presence of a mental disorder and any pre-existing psychopathology within the family.

Two other causes may explain the elevated prevalence rates over time. First, the way in which the Ghislenghien disaster was managed in the immediate aftermath; for example, unclear, confusing or inaccurate information was given at an early stage of the event. This may have led to increased fear, distrust, anxiety, depression and anger [16,18]. However, it is known that human-made disasters often have an unclear endpoint and result in distrust of authorities [19-21]. Crisis management errors and a lack of adequate psychosocial assistance may have played a role in the detrimental mental health effects. Second, the fact that the responsibilities in this disaster were unclear for a long time may have leaded to ongoing pathogenic processes and to a long-lasting psychological impact among the SHD. Anecdotic evidence coming from personal contacts with victims during support meetings and answers on open-ended questions of the questionnaire indicated that this was particularly true for victims who reported exposure to severe human suffering.

### Limitations of the study

In most traumatized populations, risk for traumatic events is confounded with pre-existing psychosocial factors [22]. The design of our study does not rule out the possibility that some participants had histories of mental health problems before the disaster. We could not document the occurrence of symptoms and patterns of complaints prior to the disaster. Nevertheless, a causal assumption may be generated as the symptoms may be attributed to the traumatic event [22].

The rather low response rate of 18% at household level at T1 urges a careful interpretation of the reported prevalence rates. Indeed some response contagion may not be excluded. In addition, only 8.2% of the total study population consisted of SHD showing large confidence intervals. This suggests that other factors than the exposure type play a role in disturbing mental health. A larger number of participants would have been preferable to stabilize the estimates. The non-response could have taken place at three levels: first of all, people may not have been able to respond to the questionnaire because of hospitalization or recovery, physical or emotional. On the other hand, some may not have responded to the questionnaire because they felt they were not involved in the disaster or because they were disappointed about the government’s management of the disaster or did not feel concerned by this study.

Nevertheless, this group represents about one fourth of the total number of victims that had been injured in the disaster (23.5%, 31/132 injured victims participated). Up till now, there is no systematic investigation of the mental or physical health of the population after a disaster in Belgium. If this was the case, the chances are high that more victims would accept to participate in such an investigation because there would not be the competition between various research teams. Furthermore, it is acceptable that a clear official policy followed by a widespread information campaign about the objectives of disaster research would lead to a better response rate among victims.

The previous discussion points might indicate that the prevalence rate of PTSD in victims and their families is underestimated. Due to organizational constraints of this study, the families of the deceased victims could not be contacted since their addresses have not been made available and another study on the health consequences of this disaster has been implemented in the burn injury centers. Mental health disturbances might have been more pronounced in this group.

The somatization disorder is not listed among the classic responses to a disaster, however, non-diagnostic “somatization”, “somatization symptoms”, and “somatic symptoms” have been abundantly described in the literature [22]. This study used the SCL-90-R to assess people for somatic symptoms. The SCL-90-R has been the most popular symptom scale in post disaster psychiatric assessment [20]. The scale does not provide diagnostic information, but identifies cases of somatization through the establishment of cutoff points for caseness [23]. However, the adoption of symptom scales implies methodological shortcomings when used in trauma research. A first shortcoming of the SCL-90 is the construct validity whereby it fails to differentiate “somatic” symptoms (referring to any physical symptom/complaint with any medical basis) from “somatoform” symptoms (limited to physical symptoms without a medical basis). Second, somatization symptoms scales are unable to detect and correct for response sets and social desirability [24,25]. Third, it has been found that the use of the SCL-90-R in trauma research measures global distress and fails to differentiate somatization from depression and anxiety [26]. These shortcomings could not be corrected in this study. Nevertheless, the 4 MHD’s are interwoven. For example, anxiety can produce upset stomach, shortness of breath, sleeping difficulty, poor concentration and general agitation. Depression can also lead for example to sleep disturbances and difficulty in performing daily activities [27-30]. This is the reason why a co-morbidity index was developed and looked at its evolution over time.

### Strengths of the study

A first strength of this study was the availability of a reference population, serving as a control group. The adults older than 15 living in the province of Hainaut were chosen as an unexposed reference population for reasons of closest comparability with the study population (Ghislenghien lies within this province). This province took part in the 2001 Belgian National Health Interview Survey. However, in spite of the use of the SCL-90-R, people may have answered differently in the aftermath of a disaster due to the social impact on the public as a whole, inducing observation bias. For example a lot of attention was given to the affected population in the media. Yet, this aspect was taken into account when collecting the second wave of data by distributing the 2nd questionnaire 14 months after the disaster instead of 12 months, being a moment in time on which a lot of attention was given in the media due to the one-year commemoration of the disaster.

This study identified a group being at particular risk for adverse health effects and may help (psychosocial) crisis managers to better tailor the resources to the needs. The people directly involved who have seen human damage (SHD) are most in need for help and this help should be provided for a longer term period as the prevalence of 4 MHD does not decrease over 14 months.

## Conclusions

We believe that the present study is a valuable contribution to the disaster research. The use of a reference population, serving as an ad hoc control group, and the longitudinal design enabled a scientific measurement and description of the health effects other than the classic PTSD symptoms or symptom clusters in a cause-effect perspective. These results also provided information for policy makers concerning the type and duration of health effects in disaster-affected adults and calls attention to the fact that mental health problems are long-lasting among survivors who have been witnessing human damage. We would like to advise to re-evaluate the impact of the Ghislenghien disaster on the longer term.

### Consent

Written informed consent was obtained from the patient’s guardian/parent/next of kin for the publication of the scientific results of the Ghislenghien study and any accompanying figures, images or data.

This study was approved by the Ethical Board of the Scientific Institute of Public Health (Brussels, Belgium).
